# Chemical Profile, Antibacterial and Antioxidant Potential of *Zingiber officinale* Roscoe and *Elettaria cardamomum* (L.) Maton Essential Oils and Extracts

**DOI:** 10.3390/plants11111487

**Published:** 2022-05-31

**Authors:** Kelthoum Tarfaoui, Najiba Brhadda, Rabea Ziri, Asmaa Oubihi, Hamada Imtara, Sara Haida, Omkulthom M. Al kamaly, Asmaa Saleh, Mohammad Khalid Parvez, Saad Fettach, Mohammed Ouhssine

**Affiliations:** 1 Laboratory of Animals, Plants Production and Agro-Industry, Department of Biology, Faculty of Science, Ibn Tofail Universty, Kenitra 14000, Morocco; kelthoum.tarfaoui@gmail.com (K.T.); najiba.brhadda@uit.ac.ma (N.B.); rabea.ziri@uit.ac.ma (R.Z.); 2 Laboratory of Natural Resources and Sustainable Development, Department of Biology, Faculty of Science, Ibn Tofail University, Kénitra 14000, Morocco; oubihi.as@gmail.com (A.O.); mohammed.ouhssine@uit.ac.ma (M.O.); 3 Faculty of Arts and Sciences, Arab American University Palestine, 240, Jenin 44862, Palestine; 4 Laboratory of Separation Processes, Team of Environment and Applied Chemistry, Faculty of Sciences, Ibn Tofail University, Kenitra 14000, Morocco; sara.haida@uit.ac.ma; 5 Department of Pharmaceutical Sciences, College of Pharmacy, Princess Nourah bint Abdulrahman University, P.O. Box 84428, Riyadh 11671, Saudi Arabia; omalkmali@pnu.edu.sa (O.M.A.k.); asali@pnu.edu.sa (A.S.); 6 Department of Pharmacognosy, College of Pharmacy King Saud University, Riyadh P.O. Box 2457, Saudi Arabia; mohkhalid@ksu.edu.sa; 7 Biopharmaceutical and Toxicological Analysis Research Team, Laboratory of Pharmacology and Toxicology, Faculty of Medicine and Pharmacy, Mohammed V University, Rabat 10106, Morocco; saadfettach.mar@gmail.com

**Keywords:** *Zingiber officinale* Roscoe, *Elettaria cardamomum* (L.) Maton, essential oil, chemical composition, antibacterial properties, antioxidant activity

## Abstract

The aim of this work was to study the chemical composition of the essential oil extracted from ginger rhizomes (*Zingiber officinale* Roscoe) and cardamom seeds (*Elettaria cardamomum* (L.) Maton). Using gas chromatography coupled with mass spectrometry (GC/MS), a total of 43 compounds were identified in ginger essential oil and 17 compounds in cardamom. The most abundant components, respectively, were zingiberene (22.18%) and 1.8-cinéol (43.47%). Essential oils, methanol, ethanol and chloroform extracts for both plants were tested against nine bacteria and yeast. The highest sensitivity was noticed against *Staphylococcus aureus* with a 25 mm inhibition zone. The antioxidant potency of both oils and extracts were measured using DPPH (1,1-diphenyl-2-picryl hydrazyl) free radical scavenging and the ferric reducing power (FRP) method; the ethanolic extract of cardamom fruits exhibited the best results for both tests, with an IC 50 = 0.423 ± 0.015 mg/mL and 95.03 ± 0.076 FRP mg AAE/g.

## 1. Introduction

According to the World Health Organization (WHO), 88% of Member States have acknowledged their use of traditional medicine, which corresponds to 170 Member States [1]. Considerable economic benefits in the development of this medicine and in the use of medicinal plants for the treatment of various diseases have been observed [2]. Therefore, the search for the potential active ingredients in the plant is more relevant than ever. Essential oils, plants extracts and their components are starting to have a lot of interest as a potential source of natural bioactive molecules. They are being studied for their possible use as an alternative for the protection of food against oxidation [3,4]. Undoubtedly, chemical or synthetic food preservatives are part of the techniques and means to ensure consumer safety, extend the shelf life of food products and limit their alterations by oxidation [5]. However, the search for new molecules has proved necessary because these synthetic substances have shown a number of disadvantages and limitations of use, and they are responsible for undesirable effects [6].

Ginger and cardamom are member of the family Zingiberaceae, a small family with more than 45 genera and 800 species; their scientific names are *Zingiber officinale* Roscoe and *Elettaria cardamomum* (L.) Maton. Ginger is an erect perennial plant growing from one to three feet in height; its stem is surrounded by the sheathing bases of the two ranked leaves. A clublike spike of yellowish, purple lipped flowers that rarely flowers in cultivation. The cultivation of *Z. officinale* Roscoe is known to originate from China and then spread to India, West Africa and South East Asia [7,8]. *Zingiber officinale* Roscoe have been used for traditional medicine applications, including for the cardiovascular diseases, nervous diseases, constipation, respiratory disorders, diabetes and asthma [7,9,10]. The ginger is also known for its antiobesity [11], antidiabetic [12], anti-inflammatory [13] and digestive virtues [14].

*Elettaria cardamomum* (L.) Maton is a woody herbaceous plant of 30 cm to 3 m high, with underground rhizomes, and pseudo-stems aerial (tillers) made of leaf sheaths. It is cultivated widely in tropical regions in India, Costa Rica, Indochina, Sri Lanka, Mexico, Indonesia and Tanzania [15,16]. Studies on vegetative growth indicate that groundcover continue to grow for a period of approximately 18 months from the moment of emergence [17]. Cardamom blossoms develop on leafless shoots from the rhizome; the fruit is a capsule usually 7 mm in size with a green color, containing many highly aromatic black seeds with capsules round or long, but they all have the same taste and properties [18]. Traditional medicine has used cardamom capsules, as popular remedies to treat many conditions, cardiac and kidney disorders, teeth and gum infections, nausea, diarrhea, asthma and digestive disorders [19,20,21]. In recent years, *E. cardamomum* has been found to possess biological activities, such as gastric antiulcerogenic [22] anti-inflammatory [23], antidiarrhoeal [24], antidiabetic [25] and antimutagenic [19].

Many studies shown that the extracts and essential oils extracted from *E. cardamomum* (L.) and *Z. officinale* Roscoe have antioxidant activities that are capable to preventing oxidative damage and infection diseases [26,27,28]. These activities are due to the fact that these plants contain many phenolic compounds and biologically active compounds, such as gingerol in the extract from *Z. officinale* and β-zingiberene, geraniol, neral, β-bisabolene and β-sesquiphellandrene in their essential oil, while 1, 8-cineole α-terpineol, sabinene nerol and α-pinene was found in the essential oil of *E. cardamomum* (L.) [14,23,27].

Both these Zingiberaceae plants are widely used in Moroccan culinary and as traditional remedies for different illnesses. There is no in-depth study showing the antioxidant and antibacterial activities of the extracts and essential oils extracted from these plants in the Kenitra region. Therefore, the main objectives of the present work were to determine the antioxidant and antibacterial activities of extracts and essential oils extracted from *E. cardamomum* (L.) and *Z. officinale* Roscoe against resistant pathogenic bacteria and yeasts collected from the center of medical analyses of Kenitra: *Staphylococcus Aureus, Staphylococcus epidermidis, Escherichia coli, Klebsiella pneumoniae, Proteus Mirabilis, pseudomonas aeruginosa, Acinetobacter baumannii, Candida tropicalis* and *Candida albicans*. In addition, the chemical compositions of both plants were studied.

## 2. Results and Discussion

### 2.1. Chemical Composition

The analysis of the essential oil of ginger by gas chromatography–mass spectrometry (GC-MS) allowed to identify forty-three compounds (Table 1). The most abundant components in ginger were: a α-Zingiberene (22.18%), β-Sesquiphellandrenene (11.05%), 1.8-cineol (6%), Geranial (5.13%) and β-Bisabolene (4.96%). This result is in line with those published by Debbarma et al. and Sasidharan and Menon [29,30], where zingiberene is the main component, with respective rates of 27.40% and 30.3%.

Different results published announce differences of the compositions. Singh et al. showed that Geranial is the main compound found in ginger [31], while El-Baroty et al. and Srinivasan showed that the essential oil of ginger was characterized by the abundance of b-sesquiphellandrene (27.16%) [26,32]. This difference can be allocated to several factors; among them, the main factors were the variety of agro-climatic conditions (climate, season and geographical zone), stadium of maturity of the plant and the adaptive metabolism [33], in addition to the drying method and the size of the slices cut before the drying [34,35].

The main compound found in *E. cardamomum* essential oil was 1.8-cineol (43.47%) followed by α-terpinyl acetate (21.56%) and linalool (10.26%); those three compounds represent more than 70% of the oil. Those results are in line with the literature where 1.8-cineol is the most abundant component [11], while other studies refer to Terpinyl acetate as the main one [31]. The essential of *Z. officinale* was mainly composed of sesquiterpenes compounds, while the essential oil of *E. cardamomum* was monoterpene dominant.

### 2.2. Total Phenols Content

Table 2 illustrates the total phenols, flavonoids and tannins compounds of *Z. officinale* Roscoe and *E. cardamomum* (L.) Maton extracts. High TPC, TFC and TCC contents are found in the ethanolic extracts *Z. officinale* and *E. cardamomum*: 29.78 ± 1.43 mg GAE/g, 44.01 ± 2.74 mg RE/g and 6.78 ± 0.25 mg CE/g in *Z. officinale* extracts and 33.45 ± 0.35 mg GAE/g, 67.38 ± 1.03 mg RE/g and 10.25 ± 1.84 mg EC/g in *E. cardamomum* extracts. In contrast, the chloroformic and methanolic extracts Has slightly lower levels of TPC and TFC; however, the TCC is extremely low. This variation in the results clearly shows that the difference in polarity of the solvents influences the extraction of phenolic compounds [36].

These results are more interesting than those found by Chen et al., who showed that the total polyphenol contents of the rhizome extracts of *Zingiber oligophyllum* and *Zingiber kawagoii Hayata* are, respectively, around 20 and 28 mg GAE g-1 MS [37]. On the other hand, the results found by Oueslati et al. are more relevant than our results, as the extracts were found to contain polyphenol contents almost twice those obtained with contents in the range of 51.7 mg GAE g-1 M [38]. The variations observed in the content of secondary metabolites can be explained by variations in the method of extraction and the method of preservation of the extracts [39], the harvest period and, thus, the method of drying the plant and the climatic conditions [40,41].

### 2.3. Antibacterial Activity

The essential oils of *Z. officinale* and *E. cardamomum* showed a significant antibacterial activity on the tested bacteria (Table 3). However, the studied bacteria did not demonstrate the same sensitivity towards the essential oils. The highest sensitivity, 24 mm, was recorded for *Staphylococcus aureus* by *Z. officinale*, and 20 mm by *E. cardamomum*. The inhibition zones on *Staphylococcus epidermidis* are 13 mm and 14 mm. These results were in accordance with previous reports indicating that Gram-positive bacteria are less resistant to essential oils compared to Gram-negative bacteria [42]. The oil showed a better potency compared to the antibiotics, since *Staphylococcus* is resistant to Ampicillin and shows a 12 mm diameter against Amoxicillin. The effect of *Zingiber officinale* Roscoe essential oil against *Staphylococcus* is reported in several studies [43]. *Proteus mirabilis* and *Acinetobacter baumannii*, which were resistant to antibiotics, showed the same sensitivity towards the essential oils with a 7 mm diameter. *Klebsiella pneumoniae* and *Pseudomonas aeruginosa* are resistant to both essential oils.

*E. cardamomum* EO and *Z. officinale* EO showed almost similar effect on *Candida albicans* and *Candida tropicalis* with a 13 mm and 12 mm zone of inhibition, respectively [30].

Ethanol extracts for both plants exhibited a moderate activity against *Staphylococcus aureus*, *Staphylococcus epidermidis* and *Klebsiella pneumoniae*, with an inhibition zone ranging between 8 to 10 mm, which is considered better than Ampicillin. Similar results regarding *Z. officinale* ethanolic extract were reported by Oueslati et al., showing that the extracts had no antibacterial activity against *Staphylococcus aureus* and *Escherichia coli*; nevertheless, bacterium *Enterococcus faecalis* was sensitive to the extract and showed an inhibition of (10 mm) [38].

Methanol extract of *Z. officinale* shows an activity against *Staphylococcus aureus* and *Staphylococcus epidermidis*, with an inhibition zone of 8 mm.

Chloroform extract of both plants exhibit an antibacterial activity against *Staphylococcus* species better than those recorded by Ampicillin.

Table 4 shows the minimal inhibitory concentration obtained by the serial dilutions method.

Up to 2 µL/mL of both oils inhibited the growth of the *Candidas*, *Staphylococcus epidermidis* and *Staphylococcus aureus*. Cardamom essential oil at a concentration of 2 µL/mL was able to hinder the growth of *Acinetobacter baumannii*.

Research has shown that essential oils rich in 1.8-cineol and poor in phenolic derivatives do not exhibit antimicrobial activity [44,45,46]. However, the results obtained demonstrate that *Z. officinale* and *E. cardamomum* essential oils have antibacterial activity on the majority of strains studied despite their high 1.8-cineol content. The antibacterial activity may be the result of the presence of α-pinene, α-terpineol and linalool, compounds that are known to possess antibacterial activity [47]. Although present in low concentrations, those constituents could have imparted a significant effect on the antibacterial activities of the oils via a synergistic effect [48].

### 2.4. Antioxidant Activity

The evaluation of the antioxidant activity of the species *Zingiber officinale* Roscoe and *Elettaria cardamomum* (L.) Maton was carried out, using two different techniques, DPPH and FRP.

The results of DPPH test show a dose dependent activity that can be evaluated by determination of IC_50_ values. Lower values of IC_50_ indicate a stronger antioxidant activity. Table 5 shows that the *Z. officinale* ethanol extract (IC_50_ = 0.712 ± 0.012 mg/mL) was significantly more potent than the *E. cardamomum* extract (IC_50_ = 0.423 ± 0.015 mg/mL). However, methanol extract *Z. officinale* (IC_50_ = 0.930 ± 0.082 mg/mL) have not shown a significant antioxidant activity compared to methanolic extract of *E. cardamomum* (IC_50_ = 0.731 ± 0.103 mg/mL). IC_50_ of *Z. officinale* chloroform extract and essential oil are close to each other with an IC_50_ = 1.218 ± 0.130 mg/mL for the first and IC_50_ = 1.298 ± 0.002 mg/mL for the second. *E. cardamomum* essential oil noted the highest IC_50_ (IC_50_ = 1.429 ± 0 mg/mL).

*Z. officinale* and *E. cardamomum* reveal an important antioxidant activity in the FRP test (Table 4). The ethanol extract showed the highest antioxidant ability in this method as well with 95.03 ± 0.076 mg AAE/g for *E. cardamomum* followed by 94.5 ± 0.09 mg AAE/g for *Z. officinale*, followed by the methanol extract (85.76 ± 0.03 mg AAE/g, 88.12 ± 0.11 mg AAE/g, respectively). Chloroform extracts for both plants exhibited the lowest reducing power, i.e., 14.08 ± 0.042 mg AAE/g for *E. cardamomum* and 13.22 ± 0.05 mg AAE/g for *Z. officinale*. The essential oils’ reducing power are weak compared to the methanol and ethanol extract, with a value of 16.7 ± 0.1 mg AAE/g for *E. cardamomum* and 31.1 ± 2.1 mg AAE/g for *Z. officinale*. A significant positive correlation between the phenolic compounds and FRP, while a strong negative correlation between phenolic compounds and DPPH (Table 6).

Comparing the results with the other studies is, however, not pertinent; firstly, because the antioxidant content is strongly influenced by the type of solvent used. Secondly, the results are expressed in caffeine equivalents or Trolox equivalents, which makes the results not directly comparable [49].

In these tests, *Zingiber officinale* Roscoe and *Elettaria cardamomum* (L.) Maton reveal minor differences between the two plants antioxidant potential, the ethanol extracts exhibit the highest antioxidant activity in both methods could be explained by its richness in secondary metabolites. There is, indeed, a correlation between the antioxidant activity and content of phenolic compounds [50,51]. On the other hand, many studies have reported that phenolic compounds are often known by their antioxidant activity [52,53].

The present study suggests that ethanolic extract isolated from *Zingiber officinale* Roscoe is a promising for the in depth study of its protective effects against increased intrinsic antiradical (oxidative stress), and this is essentially made to the high content of phenolic constituents (gingerols, shogaols, paradols and zingerone) [54]. Similarly, the ethanolic extract of *Elettaria cardamomum* (L.) Maton reported to contain a high amount of polyphenolic compounds, such as gallic acid, tannic acid, caffeic acid and 4.5-dicaffeoyl quinic acid [55]. In another study, it was noted that the ethanolic extract of cardamom consists of epicatechin, vanillin, *p*-coumaric acid, *trans*-ferulic acid and ellagic acid, which explains the high antioxidant potent [56].

## 3. Materials and Methods

### 3.1. Plant Material

The Rhizomes of *Zingiber officinale* Roscoe and *Elettaria cardamomum* (L.) Maton seeds were both bought from the local Market of Kenitra; the country of origin is China. The voucher specimen numbers are, respectively, 798372-1 and 796556-1 [57]. Ginger was cut up to 10 mm slices and dried in the oven at 60 °C [58].

### 3.2. Extraction Protocol

#### 3.2.1. Essential Oil

Hydro-distillation in a Clevenger type apparatus was used to extraction of the essential oils [59]. This method was accomplished by boiling 300 g of the dried rhizomes of each plant in a volume of 1000 mL of distilled water in a 2 L flask for 5–6 h. The essential oil yield was stocked at 4 °C in darkness in the presence of anhydrous sodium sulfate.

#### 3.2.2. Preparation of Crude Extracts

The extraction of methanol, ethanol and chloroform extracts from the samples was carried out by solid-liquid extraction using a Soxhlet device [49]. For each plant, an amount of 50 g dried and crushed powder was put in a cartridge, which was placed in the Soxhlet extractor; then, a flask was filled for each extraction with 300 mL methanol, ethanol and chloroform, which was placed on the flask heater. The solvent evaporated, condensed in the condenser and fell back into the Soxhlet extractor, dissolved the active ingredients and returned to the recovery flask: the operation ion was repeated several times for 24 h until the powder was completely exhausted. In the end, the solvent of each solution was evaporated using a rotary evaporator until a crude extract was obtained.

### 3.3. Chromatographic Analysis

The essential oil was analyzed using gas chromatography with flame ionization detection and gas chromatography (Trace GC ULTRA) coupled to a mass spectrum (Polaris Q MS with ion trap), equipped with a 15 m × 320 μm × 0.1 μm capillary column VB-5 (5% phenyl methylpolysiloxane). A volume of 4 µL of essential oil diluted in chloroform was injected into the column in 1/4 split mode using helium as carrier gas at 3 mL min. The vector gas used was helium; the oven temperature was programmed at 200 °C for 6 min and increased to 300 °C at a rate of 20 °C/min for 10 min. The identification was carried out using NIST 2014 MS Library [60].

### 3.4. Determination of Phenolic Content

#### 3.4.1. Total Phenol Content

The phenol content was determined using the Folin Ciocalteu reagent protocol [61]. One dose of the extracts (0.5 mL) of each plant was mixed with 2.5 mL of Folin-Ciocalteu reagent diluted in water (1:10). Then, 4 mL of 7.5% Na_2_CO_3_, (w/v) was added and incubation for 30 min at 45 °C in the dark. The solutions were read using spectrophotometer at 760 nm is taken against a blank. The total content of phenols is expressed in mg of gallic acid equivalent (GAE) per gram of extract (mg GAE/g E).

#### 3.4.2. Total Flavonoid Content

The total content of flavonoid was estimated using the aluminum chloride colorimetric protocol [49]. One dose of the extracts (0.25 mL) of each plant 0.25 mL of extract diluted with 1.25 mL of distilled water and mixed with 0.075 mL of sodium nitrite solution NaNO_3_ (5%); next, the mixture was kept at rest for 5 min. Then, 0.15 mL of aluminum chloride (10%) was added and after 6 min, 0.5 mL 1 M sodium hydroxide was added. The mixture is diluted with 0.275 mL of distilled water. The final solutions were incubated for 30 min in the dark at room temperature, and the absorbance was measured at 510 nm by a spectrophotometer. The flavonoids content is expressed in mg of rutin equivalent (RE) per gram of extract (mg RE/g E).

#### 3.4.3. Total Tannin Content

The acidic vanillin method was used to determine the total content of condensed tannins [62]. One dose of the extracts (0.5 mL) of each plant was mixed with 3 mL of vanillin-methanol solution (4%) and 1.5 mL hydrochloric acid. The solution was incubated in the dark for 15 min. The absorbance was measured by spectrophotometer at 500 nm. The content of condensed tannins is expressed in mg of catechin equivalent (CE) per gram of extract (mg CE/g E).

### 3.5. Antimicrobial Activity

#### 3.5.1. Microorganisms Studied

Microbes used were resistant pathogenic bacteria and yeasts collected from the center of medical analyses of Kenitra and are: *Staphylococcus Aureus*, *Staphylococcus epidermidis*, *Escherichia coli*, *Klebsiella pneumoniae*, *Proteus Mirabilis*, *pseudomonas aeruginosa*, *Acinetobacter baumannii*, *Candida tropicalis* and *Candida albicans*. The bacterial strains were purified by subculture on Mueller–Hinton agar and incubated for 24 h at 37 °C; yeasts were incubated for 48 h at 30 °C on a sabouraud medium.

#### 3.5.2. Disc Diffusion Assay

The antimicrobial activity of the essential oils and extracts was measured by using the disc diffusion method [63,64]. A suspension (1 McFarland) of the microorganism was spread in the Petri dish on solid medium Mueller Hinton Agar (MHA) for bacteria and chloramphenicol Sabouraud for yeasts. Filter paper disc (6 mm diameter) was impregnated by each essential oil and extract with volume is 15 μL and placed on the inoculated Petri dishes. After having remained at 4 °C for 2 h, the Petri dishes were incubated at 37 °C for 24 h (bacteria) and 30 °C for 48 h (yeasts). Ampicillin (5 μg/disc) and Amoxicillin (25 μg/disc) were used individually as positive controls for bacteria.

#### 3.5.3. Minimum Inhibitory Concentration (MIC)

The method described by Remmal et al. used to determine the minimum inhibitory concentrations (MICs) [65]. The essential oil was emulsified with 0.2% of aqueous agar solution to favor contact between the bacteria and the medium. Moreover, the organic extracts were solubilized in DMSO, a solvent known to have no antibacterial effect [66]. Dilutions of essential oils and extracts were prepared in 100 µL/mL, 40 µL/mL, 20 µL/mL, 10 µL/mL, 5 µL/mL, 3.3 µL/mL and 2 µL/mL agar solutions. The test tubes contained 13.5 mL of the nutrient agar autoclaved for 20 min at 121 °C and cooled to 45 °C. In total, 1.5 mL of each of the dilutions was added to obtain final concentrations of 10 µL/mL, 4 µL/mL, 2 µL/mL, 1 µL/mL, 0.5 µL/mL, 0.33 µL/mL and 0.2 µL/mL. The tubes were then vortexed by the vortex before being poured into Petri dishes. The cultures of the various bacterial strains were carried out by striation. Control tubes containing only the culture were seeded. The cultures were then incubated at 37 °C for 24 h. Each test was repeated three times in order to minimize the experimental error.

### 3.6. Antioxidant Activity

#### 3.6.1. Ferric Reducing Power (FRP) Assay

The ferric-reducing capacity of *Zingiber officinale* Roscoe and *Elettaria cardamomum* (L.) Maton essential oils and extracts was investigated by using the potassium ferricyanide-ferric chloride method with some modifications [67,68]. Briefly, the essential oil and extracts of *Z. officinale* and *E. cardamomum* (1 mL) were mixed individually with 2.5 mL of phosphate buffer (0.2 M, pH 6.6) and 2.5 mL of 1% potassium ferricyanide. Every mixture was then incubated at 50 °C for 20 min to reduce ferricyanide into ferrocyanide. A portion (2.5 mL) of trichloroacetic acid (10%) was added to the mixture, which was then centrifuged at 3000 rpm for 10 min. Finally, 2.5 mL of the supernatant was mixed with 2.5 mL of distilled water and 0.5 mL FeCl_3_ solution (0.1%, w/v), and the absorbance was measured at 700 nm. Increased absorbance values indicate a higher reducing power. The results were expressed as ascorbic acid equivalent per gram of product dry weight (mg AAE/g dry extract).

#### 3.6.2. DPPH Radical Scavenging Activity Assay

Radical scavenging activity of the essential oils and crude extracts was measured using the stable radical DPPH by Fettach et al. [69]. A solution of DPPH (0.2 mM) was prepared, and 0.5 mL of this solution was mixed with 2.5 mL of the sample (2 to 0.125 mg/mL). The reaction mixture was vortexed thoroughly and left in the dark at room temperature for 30 min. The absorbance of the mixture was measured at 517 nm in a spectrophotometer. Lower absorbance of the reaction mixture indicated higher free radical scavenging activity. The ascorbic acid was used as reference compound. The capability to scavenge the DPPH radical was calculated using the following equation.

Scavenging activity in this assay was expressed as IC50, which represents the concentration of the extract required to inhibit 50% of the free radical scavenging activity [70]:

DPPH scavenging effect (%) = ((A0 − A1)/A0) × 100

where A0 is the absorbance of the control reaction and A1 is the absorbance of the sample solution or standard, and the experiment was carried out in triplicate.

### 3.7. Statistical Analysis

The data are expressed as mean values ± standard deviation for each measurement and analyzed by means of analysis of variance (one-way ANOVA) followed by Tukey posttests. The statistical analysis was performed using Excel. The correlation between phenolic compounds and antioxidants was conducted using SPSS (V.26).

## 4. Conclusions

The obtained results make it possible to highlight the valorization of *Zingiber officinale* Roscoe rhizomes and *Elettaria cardamomum* (L.) Maton to fight against microbial infections. This indicates that these plants can be used for the development of alternative compounds in the treatment of infections caused by the pathogens concerned. These results could provide a solid scientific basis for the search for new compounds with important applications in the food and pharmaceutical industries, so further studies for future use against diseases due to oxidative stress are recommended. However, these two plants must first be clinically studied to ensure that their use will not cause toxic damage to human health.

## Figures and Tables

**Table 1:** Chemical Composition of *Zingiber officinale Roscoe* and *Elettaria cardamomum* (L.) Maton essential oil.

Chemical KI	Compound		Area %	
			ZO	EC
α-Pinene		936	1.23	0.57
Camphene		955	3.29	-
Sabinene		977	0.32	2.11
β-Pinene		980	1.15	0.69
1.8-cineol		1035	6.00	43.47
γ-Terpinene		1059	0.07	0.72
*Trans*-Sabinene hydrate		1067	-	0.21
*Cis*-Linalool oxide		1077	-	0.06
Terpinolene		1090	0.24	0.40
Linalool		1099	0.60	10.26
*Cis*-2-p-Menthen-1-ol		1122	-	0.20
Camphor		1145	0.09	*-*
Camphene hydrate		1150	0.14	-
Isoborneol		1158	0.12	-
Endo-Borneol		1167	1.80	-
Terpinen-4-ol		1179	0.33	4.77
Isobornylmethyl ether		1184	0.15	-
α-Terpineol		1196	1.10	6.98
Neral		1241	4.06	0.49
Linalyl acetate		1258	-	1.77
Geranial		1269	5.13	-
2-Undecanone		1292	0.19	-
α-Terpinyl acetate		1351	0.34	21.56
Geranyl acetate		1376	-	0.38
α-Copaene		1377	0.29	-
β-Elemene		1392	0.69	-
Benzeneacetaldehyde, α-methyl-4-(2-methylpropyl)		1394	0.19	-
*Cis*-α-Bergamotene		1396	0.18	-
γ-Elemene		1445	0.55	-
Aromadendrene		1461	0.30	-
Germacrene D		1477	1.12	-
γ-Muurolene		1478	3.21	-
Ar-Curcumene		1483	8.40	-
α-Zingiberene		1495	22.18	-
β-Bisabolene		1509	4.96	-
β-Sesquiphellandrene		1524	11.05	-
Elemol		1549	1.01	-
Nerolidol		1565	0.38	-
*Trans*-Sesquisabinene hydrate		1583	1.18	-
10-epi-γ-Eudesmol		1622	0.41	-
7-epi-*cis*-sesquisabinene hydrate		1623	0.64	-
α-Elemene		1624	0.79	-
Zingiberenol		1626	1.95	-
β- Eudesmol		1650	1.12	-
*Trans*-Isolongifolanone		1662	0.36	-
β-Bisabolol		1672	0.97	-
Xanthorrhizol		1684	0.21	-
Ambrial		1736	-	0.11
Farnesyl acetate		1742	0.67	-
Total identified			89.16 %	94.75 %

KI: Kovats Index; ZO: *Zingiber officinale* Roscoe; EC: *Elettaria cardamomum* (L.) Maton.

**Table 2:** Total content of phenols, flavonoids and tannins of *Zingiber officinale* Roscoe and *Elettaria cardamomum* (L.) Maton extracts.

	*Zingiber Officinale*			*Elettaria Cardamomum*		
	EE	ME	CE	EE	ME	CE
TPC (mg GAE/g extract)	29.78 ± 1.43	25.15 ± 0.23	14.41 ± 1.07	33.45 ± 0.35	31.06 ± 2.10	20.36 ± 1.25
TFC (mg RE/g extract)	44.01 ± 2.74	30.59 ± 1.41	20.27 ± 1.97	67.38 ± 1.03	58.41 ± 2.03	25.01 ± 1.61
TTC (mg CE/g extract)	6.78 ± 0.25	3.64 ± 1.10	0.19 ± 2.51	10.25 ± 1.84	8.72 ± 1.36	1.49 ± 0.91

EE: Ethanolic Extract; ME: Methanolic Extract; CE: Chloroformic Extract.

**Table 3:** Antibacterial activities of *Zingiber officinale* Roscoe and *Elettaria cardamomum* (L.) Maton essential oils and extracts.

	Inhibition Zone Diameter (mm)									
	*Zingiber officinale*				*Elettaria cardamomum*				Antibiotics	
	EO	EE	ME	CE	EO	EE	ME	CE	AML	AMP
*Staphylococcus aureus*	24 ± 0.66 ^a^	8 ± 0.57 ^a^	7 ± 00 ^a^	7 ± 0.66 ^a^	20 ± 0.44 ^a^	9 ± 0.88 ^a^	8 ± 0.5 ^a^	10 ± 0.33 ^a^	12	0
*Staphylococcus epidermidis*	13 ± 0.88 ^a^	8 ± 00 ^a^	7 ± 00 ^a^	7 ± 0.44 ^a^	14 ± 1.11	10 ± 00	0 ± 00	10 ± 0.66 ^a^	7	0
*Escherichia coli*	0 ± 00	0 ± 00	0 ± 00	0 ± 00	0 ± 00	0 ± 00	0 ± 00	0 ± 00	0	0
*klebsiella Pneumoniae*	0 ± 00	7 ± 0.44 ^a^	0 ± 00	0 ± 00	0 ± 00	7 ± 0.33 ^a^	0 ± 00	00 ± 00	14	13
*Proteus Mirabibilis*	7 ± 0.44 ^b^	0 ± 00	0 ± 00	0 ± 00	7 ± 0.44 ^b^	0 ± 00	0 ± 00	0 ± 00	0	0
*Acinetobacter baumannii*	7 ± 0.12 ^b^	0 ± 00	0 ± 00	0 ± 00	7 ± 0.66	0 ± 00	0 ± 00	0 ± 00	0	0
*Pseudomonas Aeruginosa*	0 ± 00	0 ± 00	0 ± 00	0 ± 00	0 ± 00	0 ± 00	0 ± 00	0 ± 00	0	0
*Candida albicans*	13 ± 0.44 ^b^	0 ± 00	0 ± 00	0 ± 00	13 ± 00	0 ± 00	0 ± 00	9 ± 0.33	Nt	Nt
*Candida tropicalis*	12 ± 00 ^a^	0 ± 00	0 ± 00	0 ± 00	12 ± 00	0 ± 00	0 ± 00	0 ± 00	Nt	Nt

EO: Essential Oil; EE: Ethanolic Extract; ME: Methanolic Extract; CE: Chloroformic Extract; AML: Amoxicillin; AMP: Ampicillin, Nt: non tested. Statistical comparison between *Zingiber officinale* Roscoe extracts and *Elettaria cardamomum* (L.) Maton extracts was presented (values in the same column not sharing a common letter (a to b) differ significantly at *p* ≤ 0.05).

**Table 4:** Minimal inhibitory concentration of the essential oil of *Zingiber officinale* Roscoe and *Elettaria cardamomum* (L.) Maton.

Microorganisms	CMI (µL/mL)	
BACTERIA	EC	ZO
*Staphylococcus epidermidis*	2 µL/mL	2 µL/mL
*Staphylococcus aureus*	2 µL/mL	2 µL/mL
*Acinetobacter baumannii*	4 µL/mL	2 µL/mL
*Escherichia coli*	10 µL/mL	<10 µL/mL
*Klebsiella pneumoniae*	10 µL/mL	<10 µL/mL
*Proteus mirabilis*	<10 µL/mL	<10 µL/mL
*Pseudomonas aeruginosa*	<10 µL/mL	<10 µL/mL
YEASTS		
*Candida albicans*	2 µL/mL	2 µL/mL
*Candida tropicalis*	2 µL/mL	2 µL/mL

ZO: *Zingiber officinale* Roscoe; EC: *Elettaria cardamomum* (L.) Maton.

**Table 5:** Antioxidant activity of *Zingiber officinale* Roscoe and *Eletteria cardamonum* extracts and oils.

Plant	Extracts	DPPH IC_50_ (mg/mL)	FRP mg EAA/g
*Zingiber officinale* Roscoe	EE	0.712 ± 0.012 ^a,^*	94.5 ± 0.09 ^b^
	ME	0.930 ± 0.082 ^b^	88.12 ± 0.11 ^b^
	CE	1.218 ± 0.130 ^b,^*	13.22 ± 0.05 ^b^
	EO	1.298 ± 0.002 ^b^	31.1 ± 2.1 ^a,^*
*Elettaria cardamomum* (L.) Maton	EE	0.423 ± 0.015 ^a^	95.03 ± 0.076 ^a^
	ME	0.731 ± 0.10 ^a^	85.76 ± 0.03 ^a^
	CE	1.030 ± 0.02 ^b^	14.08 ± 0.04 ^b^
	EO	1.429 ± 0.01 ^b^	16.7 ± 0.1 ^b^
Ascorbic acid		0.291 ± 0.31	-

EE: Ethanolic extract; ME: Methanolic extract; CE: Chloroformic extract; EO: Essential oil. a,b comparison of different extracts from the same plant (*p* ≤ 0.05). * comparison of extracts of the two plants extracted by the same solvent.

**Table 6:** The correlation between the phenolic compounds and antioxidant activities.

	TPC	TFC	TTC	DPPH	FRP
**TPC**	1	0.925 **	0.969 **	−0.969 **	0.904 *
**TFC**	0.925 **	1	0.987 **	−0.934 **	0.761
**TTC**	0.969 **	0.987 **	1	−0.956 **	0.845 *

∗ means the correlation is significant at the 0.05 level, ** means the correlation is significant at the 0.01 level.

## Data Availability

Not applicable.
